# Supplementary material for: H-NS is a bacterial transposon capture protein
Source: Nat Commun. 2024 Aug 20;15:7137. doi: 10.1038/s41467-024-51407-5 (PMC11335895; doi:10.1038/s41467-024-51407-5)
Supplement: Supplementary file 1 — Supplementary Information [file 41467_2024_51407_MOESM1_ESM.pdf]

Figure S1

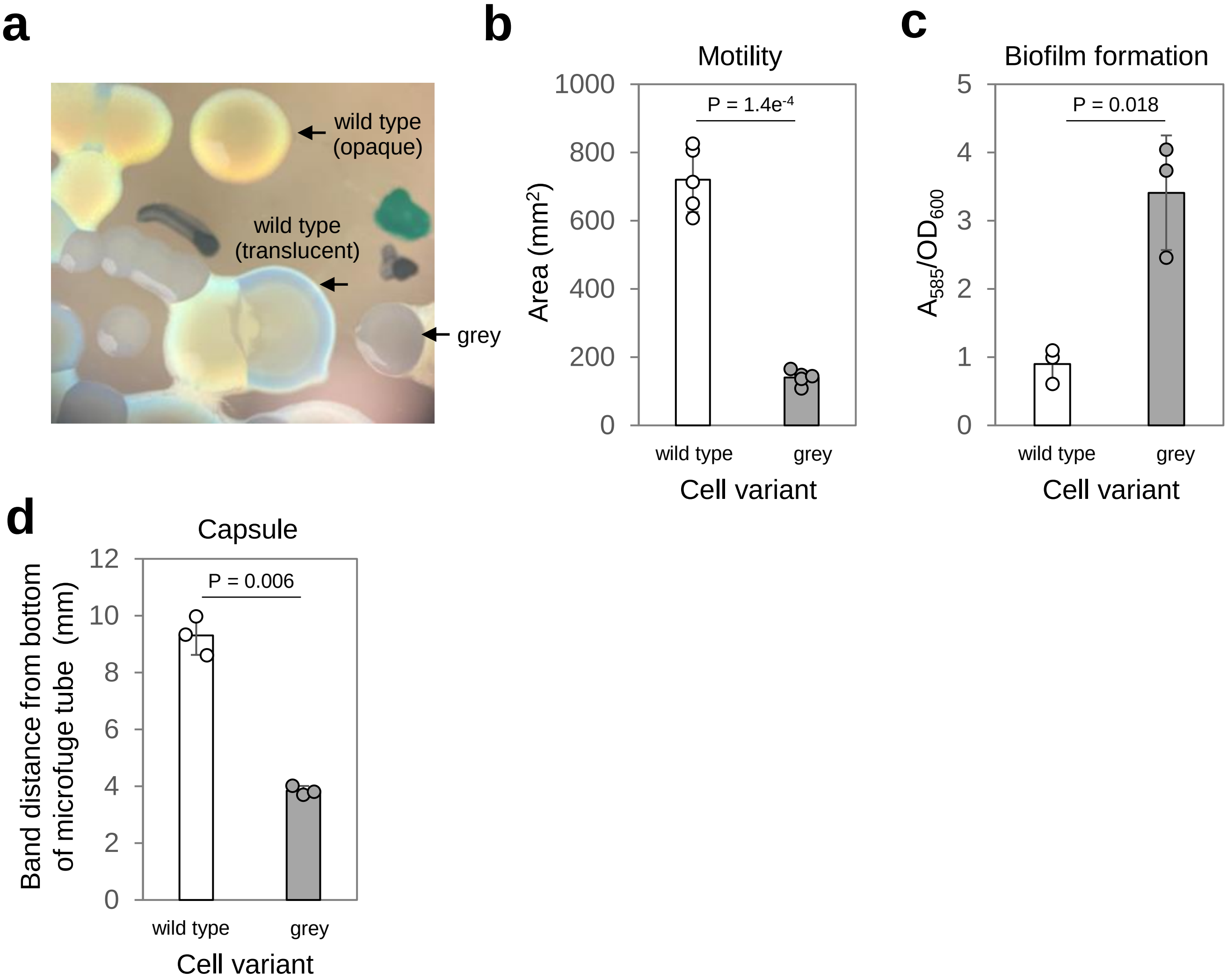

Figure S1: Phenotypic properties of grey colony variants.

a. Grey colony variants are easily distinguished by avirulent translucent colony types. A section of an agar plate viewed using a stereo microscope, illuminated from below with oblique lighting. Wild type *A. baumannii* are known to phase switch between virulent opaque and avirulent translucent colony types. These colony types, only discernible when viewed in this way, are notably different from grey colony variants that are easily identified with the naked eye.

b. Grey colony variants have reduced motility. The spread of colonies on soft agar plates was recorded photographically and the area of spread quantified. Results are the average of five biological replicates and error bars indicate standard deviation from the mean. Individual measurements are shown as discrete datapoints. A two-tailed student's t-test was used to determine P. Source data are provided as a Source Data file.

c. Grey colony variants more readily form biofilms. Cells forming biofilms on the surface of microfuge tubes were stained with crystal violet dye. After drying, the dye was solubilised the amount

present quantified by measuring the A_{585} signal as a function of the number of cells present adjudged by OD_{600} values. Results are the average of three biological replicates and error bars indicate standard deviation from the mean. Individual measurements are shown as discrete datapoints. A two-tailed student's t-test was used to determine P. Source data are provided as a Source Data file.

d. Grey colony variants are defective for capsule production. Cells separated by centrifugation in colloidal silica migrate according to the presence of capsule. Distances migrated were measured from the bottom of the microfuge tube containing the cell suspension. Results are the average of three biological replicates and error bars indicate standard deviation from the mean. Individual measurements are shown as discrete datapoints. A two-tailed student's t-test was used to determine P. Source data are provided as a Source Data file.

Figure S2

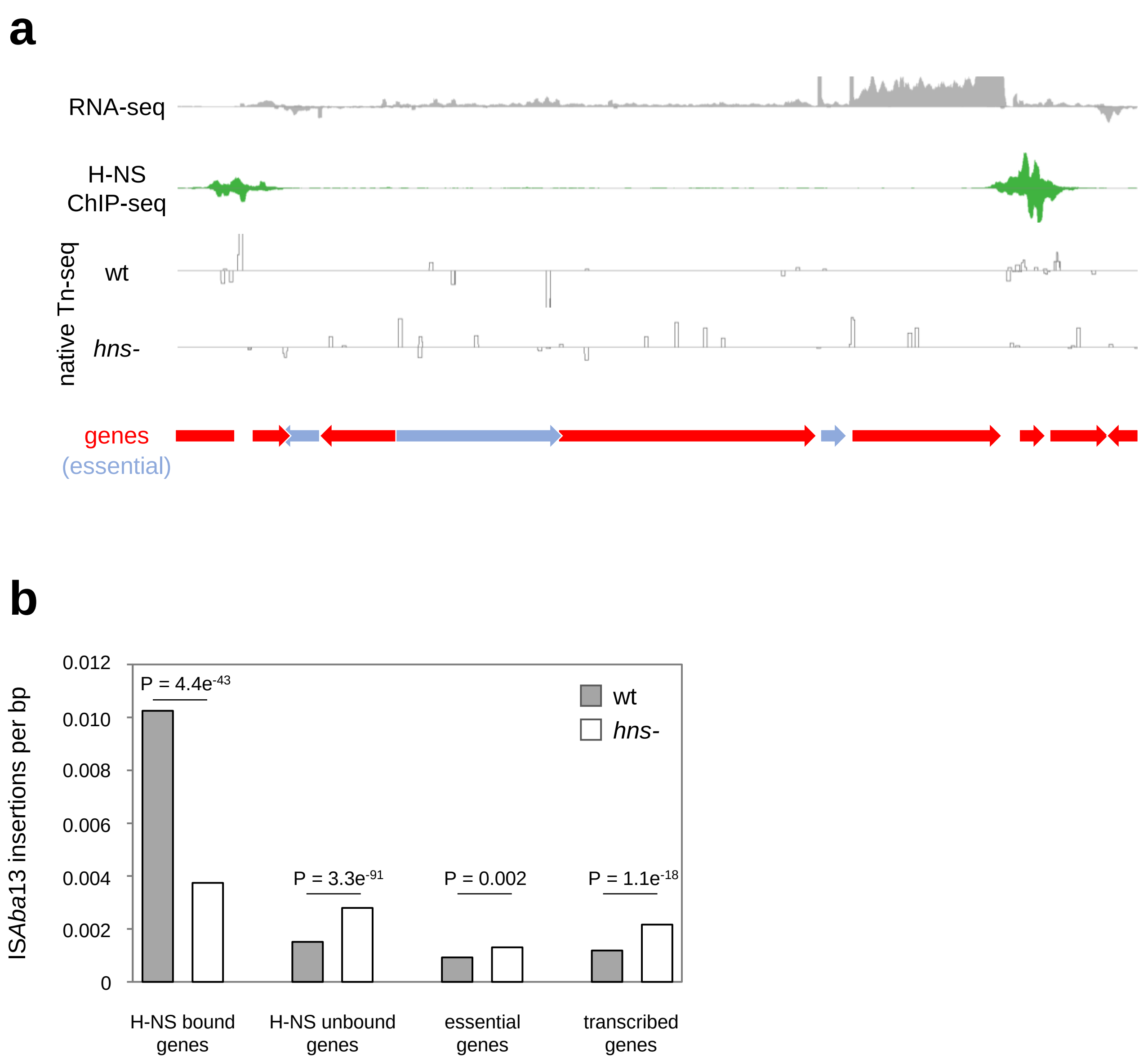

Figure S2: H-NS protects transcribed and essential genes from disruption by IS*Aba*13.

a. A representative section of the *A. baumannii* chromosome showing increased transposition into essential and transcribed genes in the absence of H-NS. Genes are shown as arrows with essential genes coloured blue. The traces show read depths from RNA-seq, H-NS ChIP-seq and native Tn-seq experiments. In all cases positive and negative values indicate read depths for the top and bottom strand respectively. The region is centred approximately around position 1,589,000 of the genome.

b. Frequency of IS*Aba*13 insertion in different classes of genes. The bar chart shows the total number of IS*Aba*13 insertions per kb, across two biological replicates, in the presence and absence of H-NS, and for H-NS bound, unbound, essential or transcribed genes. P values were derived using the student's T-test. Source data are provided as a Source Data file.

Figure S3

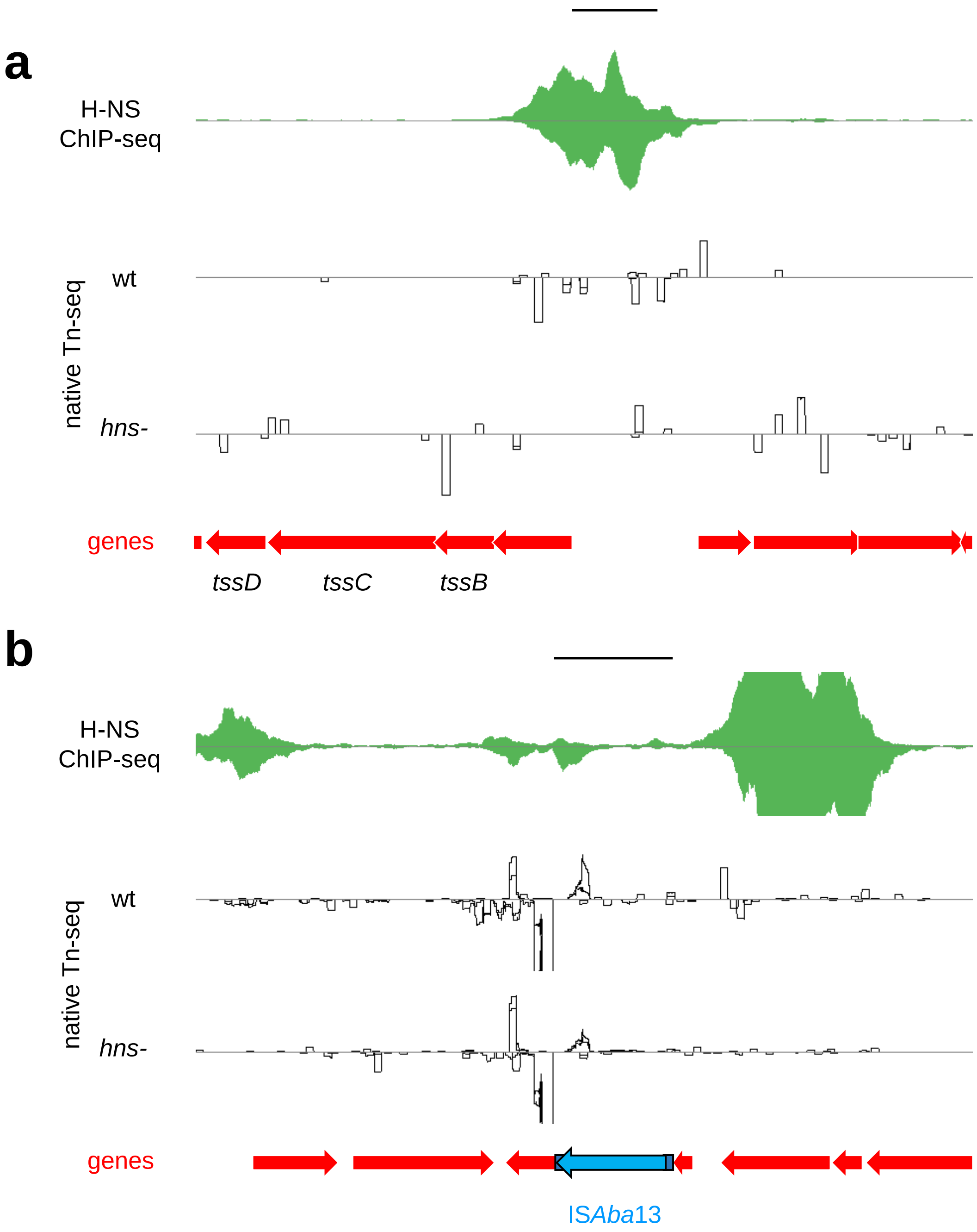

Figure S3: Patterns of H-NS binding, and IS*Aba*13 transposition, upstream of DNA regions used for *in vitro* DNA bridging assays.

a. H-NS binding and IS*Aba*13 transposition patterns at the type six secretion system encoding locus. Genes are shown as red arrows. The traces show read depths from H-NS ChIP-seq and native Tn-seq experiments. In all cases positive and negative values indicate read depths for the top and bottom strand respectively. The DNA region used for *in vitro* DNA bridging assays is indicated by the black bar.

b. H-NS binding and IS*Aba*13 transposition patterns at one of the existing chromosomal copies of IS*Aba*13. As for above panel except that IS*Aba*13 is shown in blue.

Figure S4

a

```
E. coli hns           MSEALKILNNIRTLRAQARECTLETLEEMLEKLEVVVNERREEESAAAAEVEERTRKLQQ
A. baumannii hns      -------------MKPDISELSVEELKRLQEEAEALIASKKD---------QAIEDAYNQ
                                   ::.:  * ::* *:.: *: *.:: .:::         :      :*

E. coli hns           YREMLIADGIDPNELLNSLAAVKSGTKAKRAQRPAKYSYVDENGETKTWTGQGRTPAVIK
A. baumannii hns      IIEIAENVGFSVEQLLEFGAQKRK----KTTRKSVEPRYRNKNNAEETWTGRGKQPRWLV
                        *:    *:. ::**:  *  :.    * :::..:  * ::*.  :****:*: *  :

E. coli hns           KAMDEQGKSLDDFLIKQ
A. baumannii hns      AEIEK-GAKLEDFLI--
                        ::: * .*:****
```

b

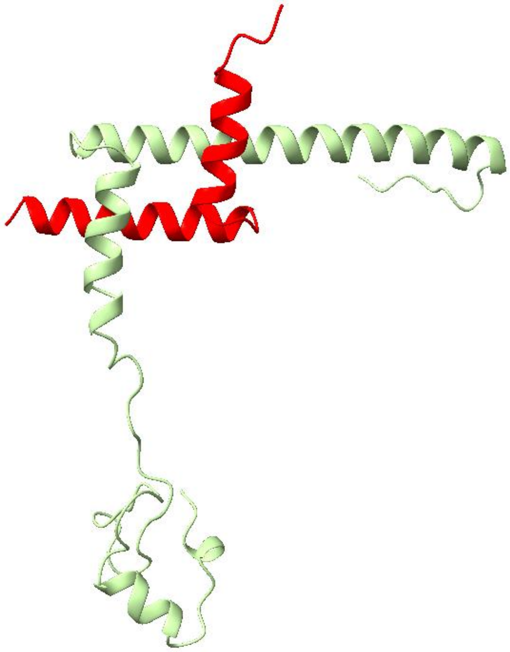

Figure S4: Organisation of H-NS-39 and predicted interaction will full length H-NS.

a. Alignment of *E. coli* and *A. baumannii* H-NS. Residues that are identical (*), conserved (:), or semi-conserved (.) are indicated. The region of *E. coli* H-NS highlighted red is the region used by van der Valk and co-workers[1]. The region of *A. baumannii* H-NS highlighted red corresponds to H-NS-39 used in this work.

b. Predicted interaction between *A. baumannii* H-NS and H-NS-39. The structural prediction was generated using AlphaFold 3[96]. Full length H-NS is green and H-NS-39 is red.

Figure S5

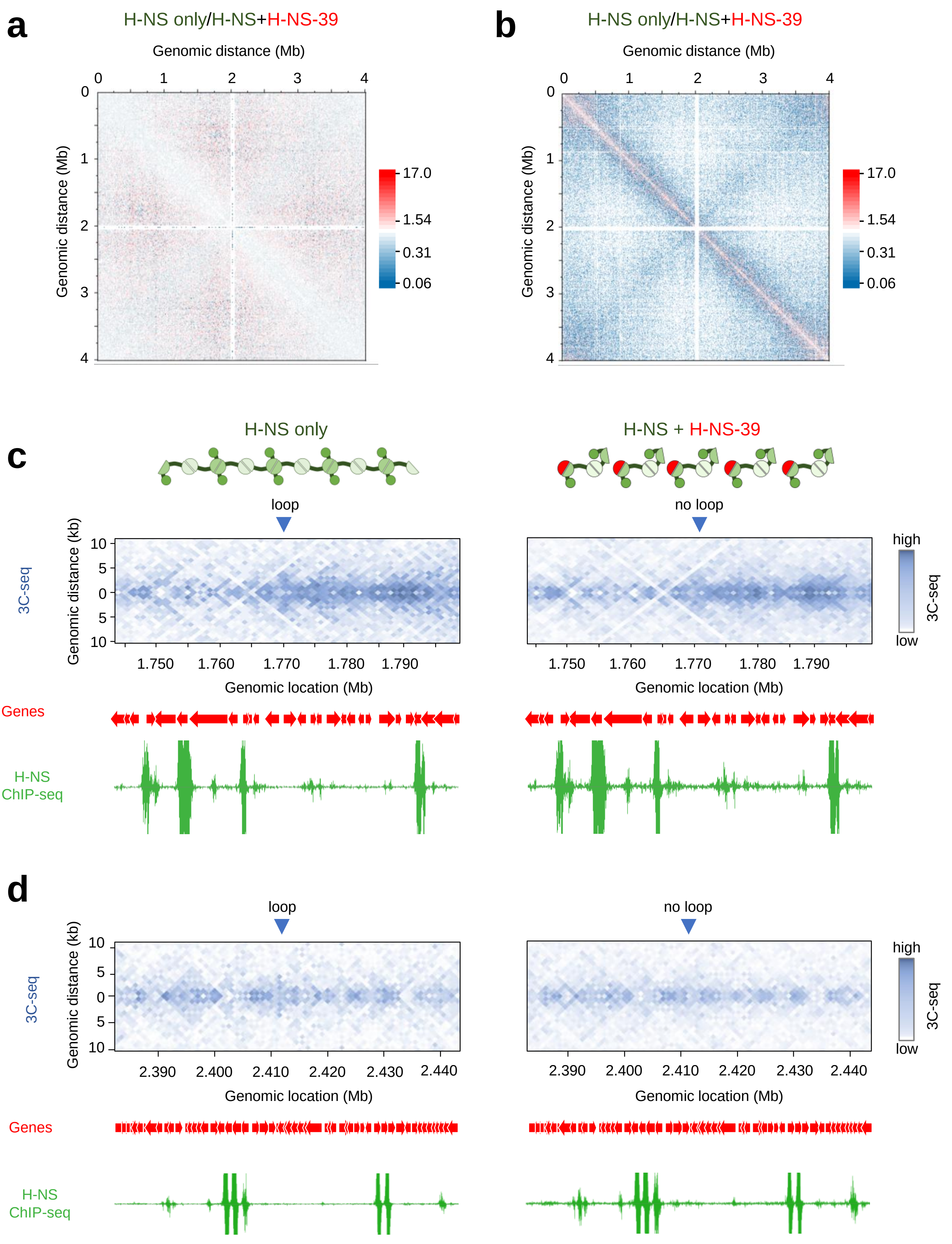
a
H-NS only/H-NS+H-NS-39
Genomic distance (Mb)
0 1 2 3 4
Genomic distance (Mb)
17.0
1.54
0.31
0.06
b
H-NS only/H-NS+H-NS-39
Genomic distance (Mb)
0 1 2 3 4
Genomic distance (Mb)
17.0
1.54
0.31
0.06
c
H-NS only
H-NS + H-NS-39
loop
no loop
3C-seq
Genomic distance (kb)
10 5 0 5 10
1.750 1.760 1.770 1.780 1.790
Genomic location (Mb)
high
low
3C-seq
Genes
H-NS ChIP-seq
d
loop
no loop
3C-seq
Genomic distance (kb)
10 5 0 5 10
2.390 2.400 2.410 2.420 2.430 2.440
Genomic location (Mb)
high
low
3C-seq
Genes
H-NS ChIP-seq

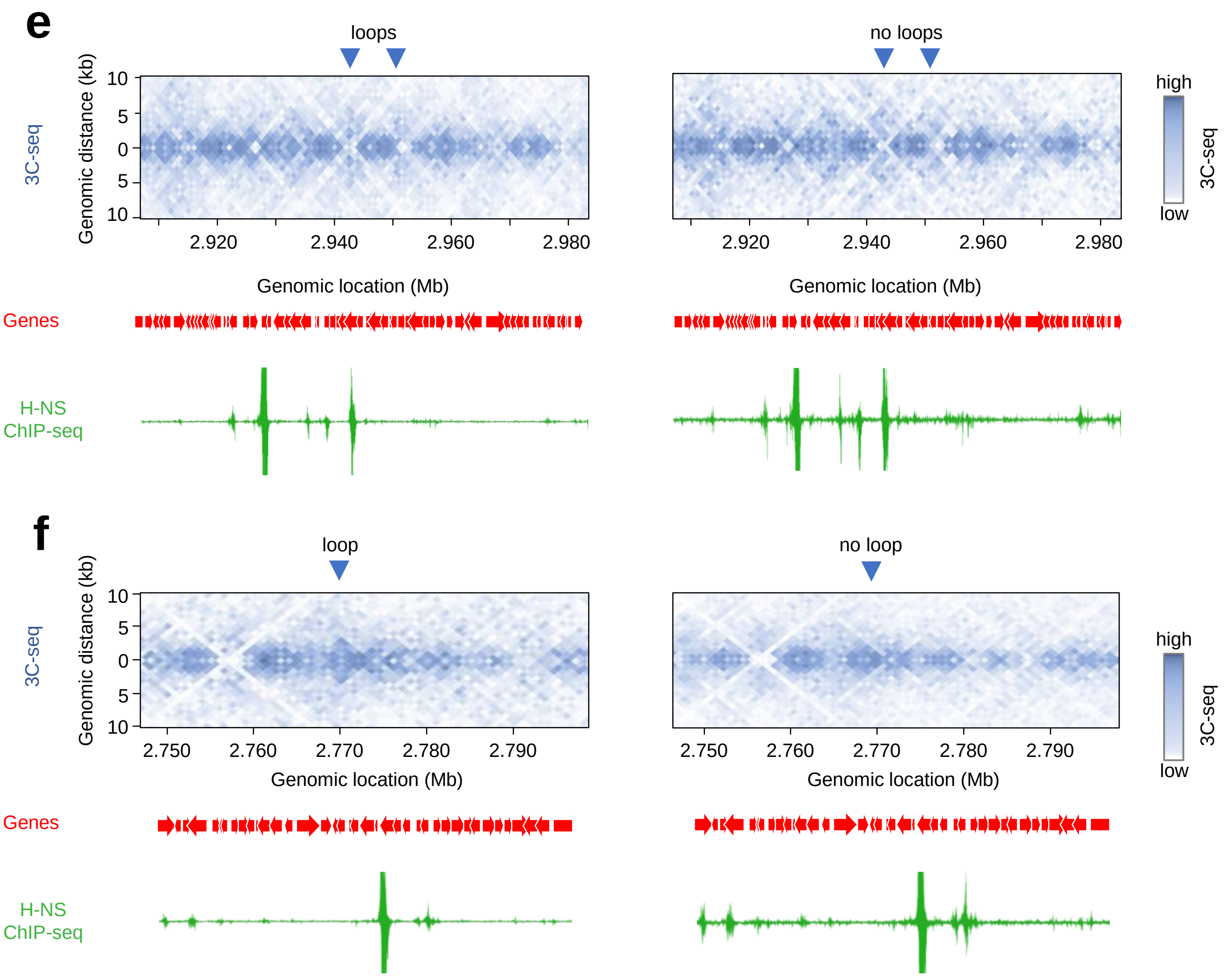

Figure S5: Disruption of local DNA folding by H-NS-39 *in vivo*.

a. Changes to 10 kb resolution contact frequencies in the presence of H-NS-39. The heatmap plots normalised 3C-seq contact frequencies, in cells without H-NS-39, divided by normalised contact frequencies from cells expressing H-NS-39. The contact ratios are grouped in 10 kb bins and axes indicate the genomic location of each bin in the pair.

b. Changes to 1 kb resolution contact frequencies in the presence of H-NS-39. As for the above panel except that values are binned at 1 kb resolution.

c-f. Changes to local DNA folding patterns in the presence of H-NS-39. In each panel, the heatmaps separately illustrate interaction frequencies between 1 kb sections of the *A. baumannii* chromosome, measured by 3C-seq, in the presence and absence of H-NS-39. An interaction pattern indicative of a loop is marked and signal in this region is lost in the presence of H-NS-39. The locations of genes (red arrows) are also shown alongside H-NS binding patterns determined by ChIP-seq with or without expression of H-NS-39.

Figure S6

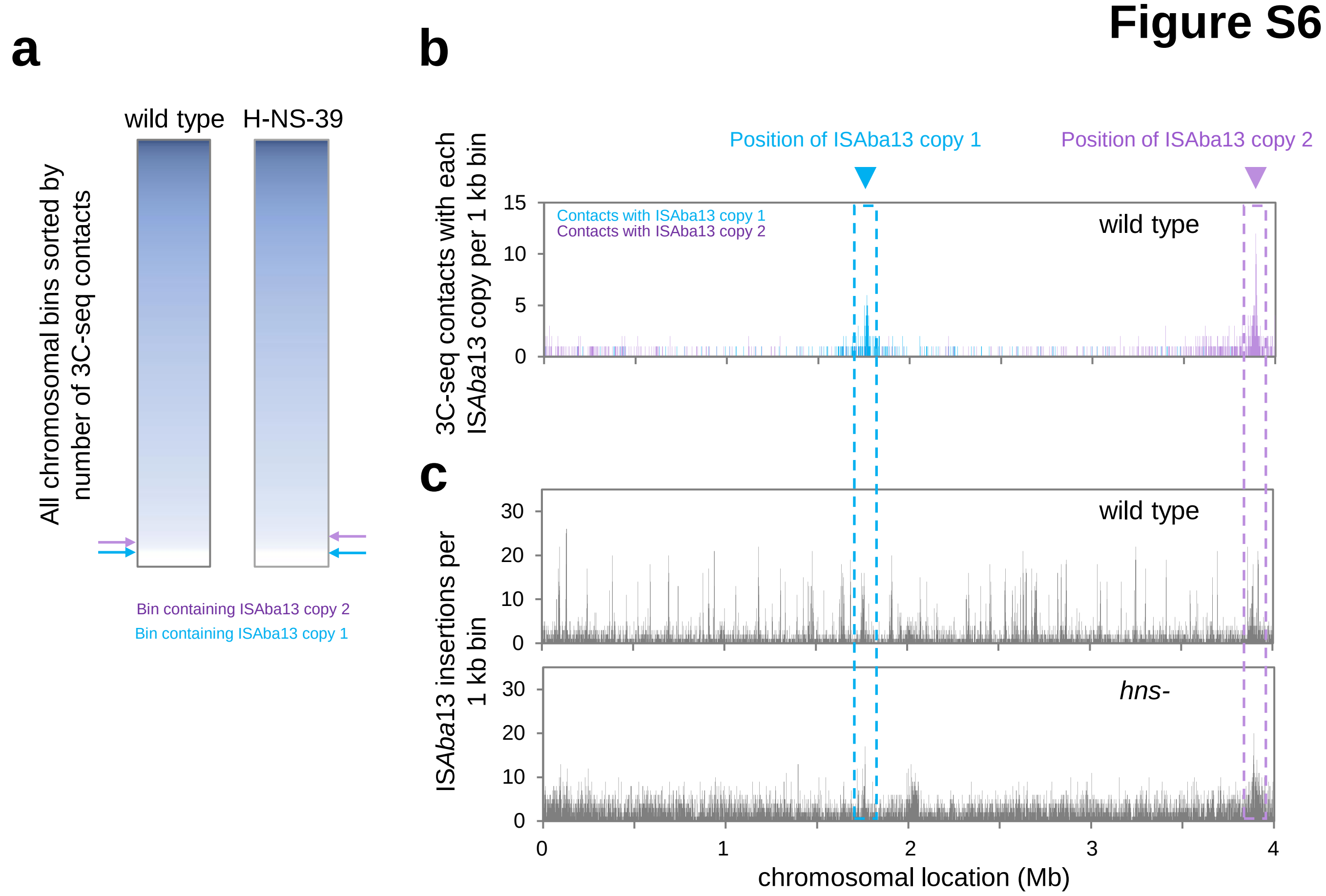

Figure S6: Existing copies of IS*Aba*13 do not interact with hot spots for IS*Aba*13 transposition.

a. Existing copies of IS*Aba*13 are in poorly interactive regions of the chromosome. The panel shows two heatmaps, each representing the *A. baumannii* chromosome divided into 1 kb sections. Sections are coloured according to the number of 3C-seq interactions and sorted on this basis, rather than by chromosomal position. Sections containing existing copies of IS*Aba*13 are labelled.

b. Location and frequency of 3C-seq interactions involving IS*Aba*13 at chromosomal positions 1.764 Mb (copy 1) and 3.863 Mb (copy 2). The bar chart shows the frequency of interactions between each 1 kb chromosomal section and IS*Aba*13 copy 1 (cyan) or copy 2 (purple). Each IS*Aba*13 copy primarily interacts with neighbouring DNA regions but not sites elsewhere in the genome. Source data are provided as a Source Data file.

c. Location and frequency of IS*Aba*13 insertions in the presence and absence of H-NS. The bar charts show the frequency of IS*Aba*13 insertions, in each 1 kb chromosomal bin, in the presence and absence of H-NS. In wild type cells, there are many hotspots for transposition that are H-NS bound regions. Comparison with panel b shows that these transposition hotspots do not interact with either copy of IS*Aba*13. In the absence of H-NS, the two regions of highest IS*Aba*13 transposition surround copies 1 and 2 of the insertion sequence at their starting chromosomal loci. Source data are provided as a Source Data file.

Figure S7

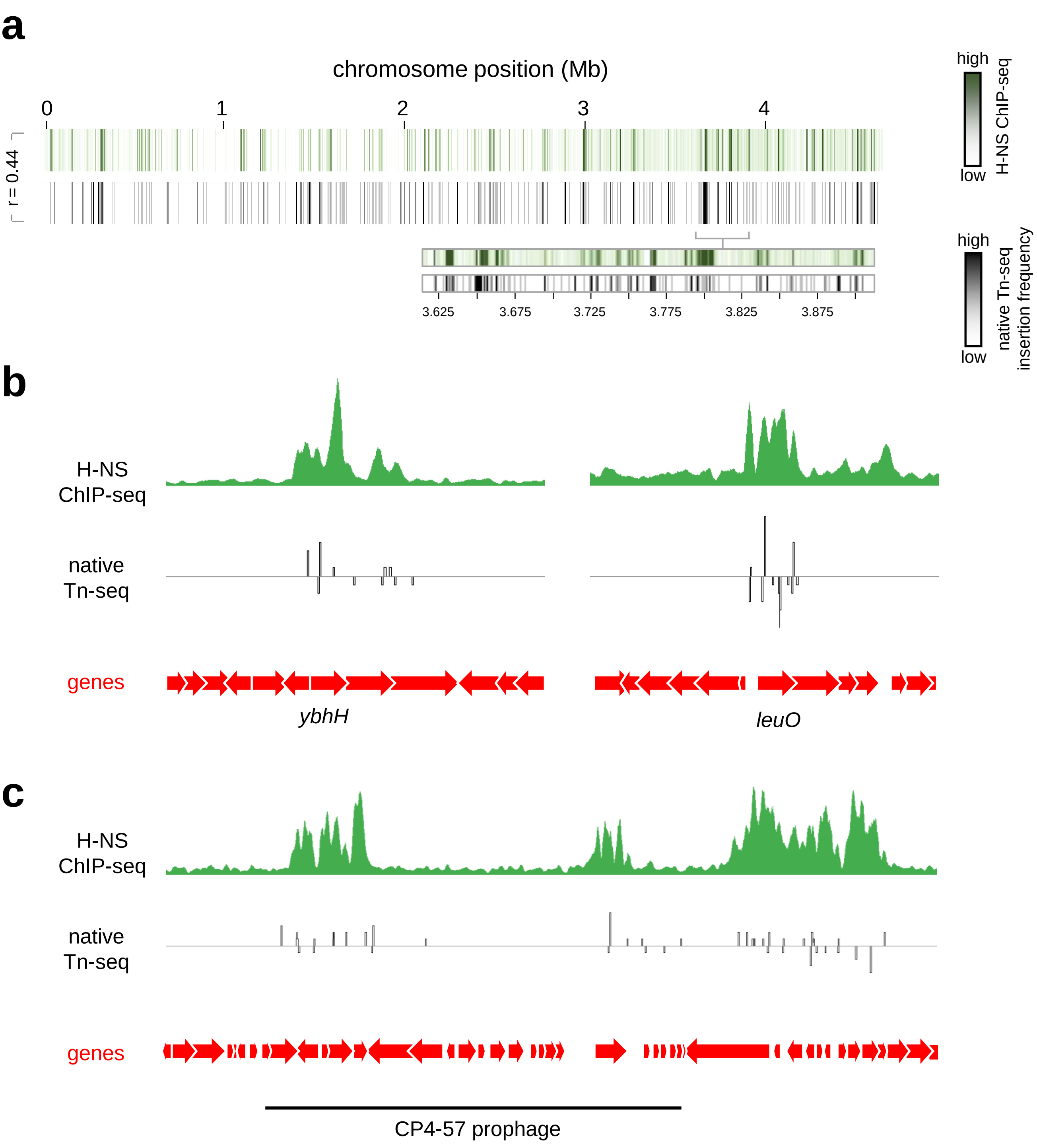

Figure S7: Insertion sequence *insH3* is targeted to H-NS bound DNA in *E. coli*.

a. Global patterns of H-NS binding and *insH3* transposition are correlated. The panel shows two heatmaps, each representing the *E. coli* MG1655 chromosome divided into 1 kb sections. Sections are coloured according to the H-NS ChIP-seq binding signal (top) or the number of *insH3* insertions detected using native Tn-seq for wild type (bottom). The heatmap expansions are provided to aid comparison of H-NS binding and insertion frequency. The Pearson correlation coefficient (r) of the two datasets is shown.

b. Examples of H-NS mediated *insH3* capture. Selected chromosomal regions with H-NS ChIP-seq and native Tn-seq data shown. In both cases, traces indicate read depths with positive and negative values corresponding to the top and bottom DNA strand respectively. Genes are shown as arrows.

c. H-NS targets *insH3* to prophage in *E. coli*. The panel shows the chromosomal region encompassing the CP4-57 prophage (marked by a solid black line). The phage genome contains two H-NS bound regions and both are targeted by *insH3*.

Figure S8

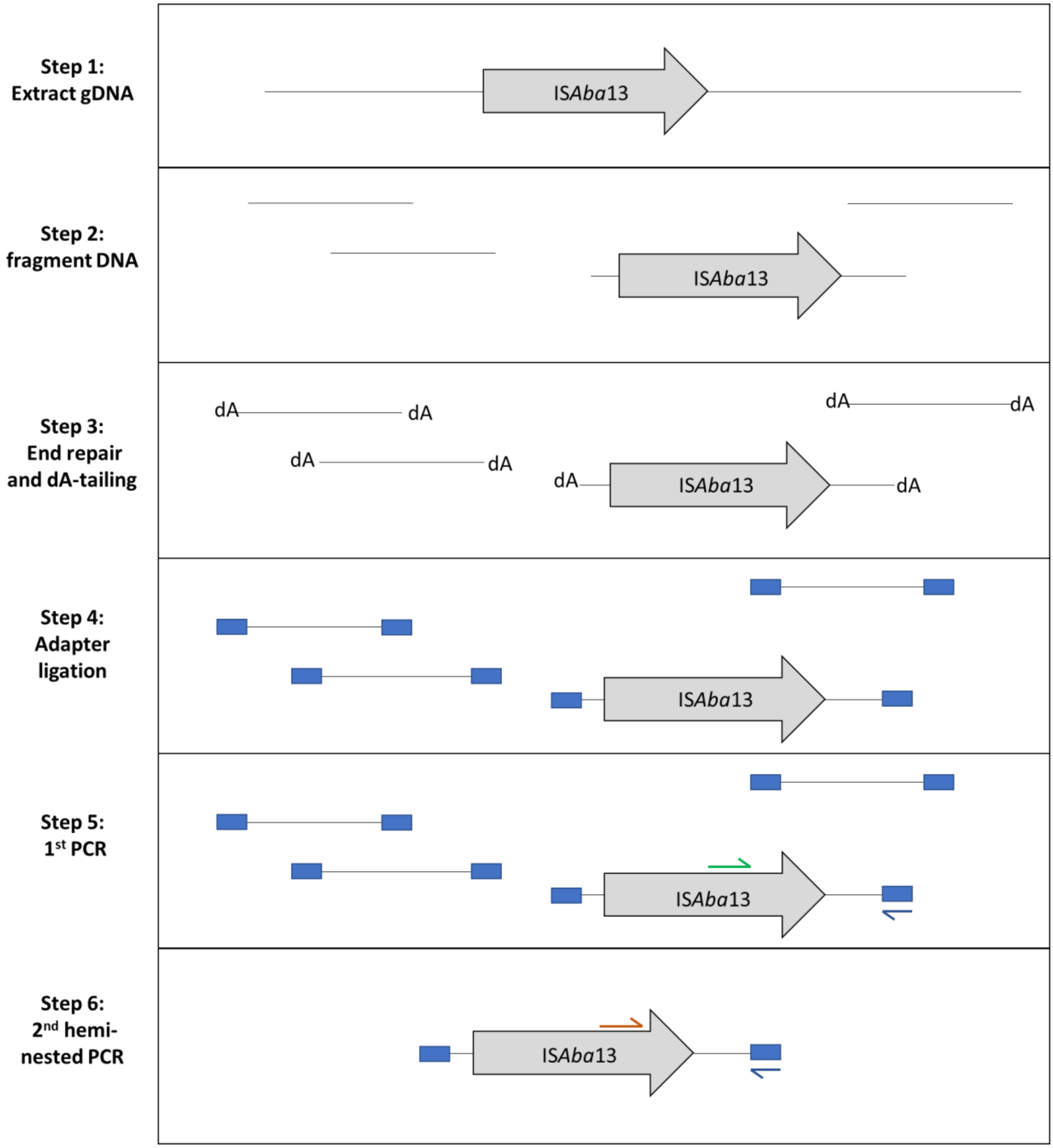

Figure S8: Schematic representation of the native Tn-seq method. Genomic DNA (gDNA) is sheared using treatment with dsDNA fragmentase. Following end repair and dA-tailing, adaptors are ligated on. This is followed by a PCR using a primer specific for the adaptor (blue arrow) and a primer complementary to the end of ISAba13 (green arrow). To increase specificity, a second hemi-nested PCR is done with the same adaptor primer and a primer complementary to IS*Aba*13 further downstream from the previous primer binding site. This is to ensure that only genuine IS*Aba*13::chromosome junctions are detected.

Table S1: Changes in gene expression in grey colony derivatives compared to wild types cells

Locus	Name	Function	Log_2 fold change	P value[1]
		Up regulated genes		
ABUW_1703		hypothetical protein	2.41	9.21E-03
ABUW_2112		DUF2726 domain protein	2.41	2.29E-02
ABUW_1766	IS*Aba*13	insertion sequence	2.14	1.14E-09
ABUW_3803	IS*Aba*13	insertion sequence	2.07	3.78E-09
		Down regulated genes		
ABUW_2006		major capsid protein	-2.12	5.09E-06
ABUW_2017		major capsid protein	-2.25	1.87E-04
ABUW_2028		major capsid protein	-2.54	2.80E-07
ABUW_1769		PEGA domain protein	-2.80	2.37E-06
ABUW_3823	*weeI*	sugar transferase	-2.94	1.82E-11
ABUW_1641		hypothetical protein	-3.25	1.35E-05
ABUW_3822	*weeH*	acetyl transferase	-3.43	2.08E-13
ABUW_0304	*pilA*	pilin	-5.49	1.22E-31

[1]Calculated using an exact test.

Table S2: Mobile genetic elements containing transposition hotspots dependent on H-NS

**Mobile element**	**Chromosomal position**	**Genes with H-NS dependent transposition hotspots**
	Chromosomal elements	
Aeromonas phage SW69-9	318756-329326	0
Escherichia phage vB_EcoM_ECO1230-10	545886-581029	1
Acinetobacter phage Bphi-B1251	736407-769903	3
Acinetobacter phage Bphi-B1251	776101-793726	0
Acinetobacter phage Bphi-B1251	1289765-1330967	6
Acinetobacter phage Bphi-B1251	1314667-1338244	2
Acinetobacter phage Ab105-2phi	1391199-1409865	0
Acinetobacter phage Ab105-2phi	1393236-1416423	0
Enterobacteria phage Ike	1985246-2029746	1
Acinetobacter phage Ab105-1phi	2650307-2669540	7
Moraxella phage Mcat16	3031838-3049068	1
	Extra chromosomal elements	
83.61 kb plasmid	N.A.	18
8.73 kb plasmid	N.A.	2
1.97 kb plasmid	N.A.	0

Table S3: Strains and plasmids

Name	Description	Source
	***A. baumannii* strains**	
AB5075	Highly virulent and drug resistant isolate from an osteomyelitis tibial infection.	(3)
AB5075::*gtr52*::IS*Aba*13	Naturally occurring "grey" AB5075 derivative with an insertion sequence disrupting the *gtr52* gene.	This work
AB5075::*ompW*::IS*Aba*13	Derivative of AB5075 with an insertion sequence disrupting the *ompW* gene, generated by scarless genome editing.	This work
AB5075 *hns*::T26	Derivative of AB5075 with a T26 transposon inserted at position 211of *hns*. Tet^R.	(4)
	***E. coli* strains**	
JCB387	Used for general plasmid DNA manipulation. Δ*nirB*Δ*lac*.	(5)
DH5α	Used for general plasmid DNA manipulation. *fhuA*2Δ(*argF-lacZ*)U169 *phoA glnV*44 Φ80Δ(*lacZ*)M15 *gyrA*96 *recA*1 *relA*1 *endA*1 *thi*-1 *hsdR*17.	NEB
T7 Express	Used to overexpress *A. baumannii* H-NS from pJ414.	NEB
MG1655	Used for native Tn-seq analysis of *insH3*.	(6)
	**Plasmids**	
pVRL1Z	High copy number plasmid, contains *parE*2-*paaA*2 toxin-antitoxin system. Zeo^R. Used to express H-NS-39 in *A. baumannii*.	(7)
pVLR2Z	High copy number plasmid, contains *parE*2-*paaA*2 toxin-antitoxin system. Zeo^R. Has an arabinose inducible promoter.	(7)
pMHL-2	Template for PCR used to make DNA fragments for genome editing. Contains *apra*R::*sacB* counter selection and resistance cassette.	(8)
pJ414	Used to overexpress *A. baumannii* H-NS in *E. coli*.	ATUM

Table S4: Oligonucleotides

Name	Description	Sequence (5' to 3')
	**for generation of *ompW*::IS*Aba*13**	
P5	To amplify *apraR*::*sacB* cassette	cgactcactatagggcgaattgggccgctttccagtcgggaaacctg
P6	To amplify *apraR*::*sacB* cassette	catatgccaccgacccgagcaaaccccgccagggttttcccagtcacgac
P62	To create fragment 1	cagcagtcacataatagatagc
P63	To create fragment 1	ggcccaattcgccctatagtgagtcgattggcaagtaaaatttggg
P64	To create fragment 2	gggtttgctcgggtcggtggcatatgggctttgttgcacaaagatttaaaag
P65	To create fragment 2	attggcaagggctttgttgcacaaacctatctc
P66	To create fragment 3	caacaaagcccttgccaattaccagca
P71	To create fragment 3	cgttatgcgcaatgtccagt
P70	To amplify Gibson assembly product	taagccatcaagcaaagtgag
P74	To amplify Gibson assembly product and create fragment used in second recombination	ctcagagctaataagtgactg
P72	For creating fragment used in second recombination	ccgtactaccttctacacggt
P157	For creating fragment used in second recombination	acttgccaatggctttgttgcacaaagatttaaaagttaag
P158	For creating fragment used in second recombination	caacaaagccattggcaagtaaaatttggg
	**for generation of *hns*-3x-FLAG**	
prSL1	To create fragments 1 and 5	ggctattaattgctgagcaagctttg
prSL11	To create fragment 2	ggtgcaaaacttgaagatttcttaatcgctactgactacaaagaccatgac
prSL12	To create fragment 2	gactgggaaaaccctggcgttacttgtcatcgtcatccttgtaatcg
prSL13	To create fragment 1	caccgtcatggtctttgtagtcagtagcgattaagaaatcttcaagttttgcacc
prSL14	To create fragment 3	cgattacaaggatgacgatgacaagtaacgccagggttttcccagtc
prSL23	To create fragment 6	gattacaaggatgacgatgacaagtaattgtattgcctcttaaaaagccaagcg
prSL24	To create fragment 5	cgcttggctttttaagaggcaatacaattacttgtcatcgtcatccttgtaatc
prSL3	To create fragment 4	ggtttcccgactggaaagcgttgtattgcctcttaaaaagccaagcgattc
prSL4	To create frarments 4 and 5	gtggacgttgatgattcaataaagcc
prSL6	To create fragment 3	cgcttggctttttaagaggcaatacaacgctttccagtcgggaaacc
prSL9	To amplify Gibson assembly product	gcaactagccaacaactcaaaaacc
prSL10	To amplify Gibson assembly product	gttgtatggtcatcacttgatcaccac
	**for cloning *A. baumannii hns* in pJ414**	
P129	To amplify codon optimised AB5075 *hns* for cloning in pJ414. *Nde*I restriction site underlined.	gcaagccatatgaaaccggacattagc
P146	To amplify codon optimised AB5075 *hns* for cloning in pJ414. *Xho*I restriction site underlined.	gcaggtctcgagttaaatcaggaaatc
	**for constructing *hns*-39 and cloning in pVRL1Z**	
P134	To create fragment 1, *Xho*I site underlined	cccccctcgagataaatattaagaaaatatattacaattataattactaatg
P135	To create fragment 1	tgatctttttcattaataaatactccagtcttac
P136	To create fragment 2	tttattaatgaaaaaagatcaagcaatcg
P137	To create fragment 2, *Pst*I underlined	cgggctgcagttatgttgttttcttacgtttttg
	**for *in vitro* DNA bridging assays**	
P162	To create IS*Aba*13 bait fragment	biotin-cttattaaatggctttgttgcac
P163	To create IS*Aba*13 bait fragment	taatttaataaggctttgttgcac

P171	To create T6SS prey fragment	caacacaactttcattcc
P172	To create T6SS prey fragment	agggtatctatatcagcca

for native Tn-seq with *A. baumannii*

Adapter 1.2	Anneals with adapter 2.2	taccacgacca-NH_2
Adapter 2.2	Anneals with adapter 1.2	atgatggccggtggatttgtgtggtcgtggtat
JelAP1	For 1st PCR, binds adapter 2.2 sequence	atgatggccggtggatttgtg
IS*Aba*13_out	For 1st PCR, binds near to the 5'end of IS*Aba*13	caaagccaagtcaatgagattcatgc
Staggered_1	For 2nd hemi-nested PCR of P5 end, binding site to IS*Aba*13 underlined. T in italic added for heterogeneity.	aatgatacggcgaccaccgagatctacactctttccctacacgacgctcttccgatc*t*gaccacatacccgagttgtcac
Staggered_2	For 2nd hemi-nested PCR of P5 end, binding site to IS*Aba*13 underlined. Ts in italic added for heterogeneity.	aatgatacggcgaccaccgagatctacactctttccctacacgacgctcttccgatc*tt*gaccacatacccgagttgtcac
Staggered_3	For 2nd hemi-nested PCR of P5 end, binding site to IS*Aba*13 underlined. TGATA in italic added for heterogeneity.	aatgatacggcgaccaccgagatctacactctttccctacacgacgctcttccgatc*tgata*gaccacatacccgagttgtcac
Staggered_4	For 2nd hemi-nested PCR of P5 end, binding site to IS*Aba*13 underlined. TATCTA in italic added for heterogeneity.	aatgatacggcgaccaccgagatctacactctttccctacacgacgctcttccgatc*tatcta*gaccacatacccgagttgtcac
AP1_P7_tagged_1	For 2nd hemi-nested PCR of P7 end, binding site to adaptor underlined. Barcode: CGTGAT in italic.	caagcagaagacggcatacgagat*atcacg*gtgactggagttcagacgtgtgctcttccgatctgtcaatgatggccggtggatttgtg
AP1_P7_tagged_2	For 2nd hemi-nested PCR of P7 end, binding site to adaptor underlined. Barcode: ACATCG in italic.	caagcagaagacggcatacgagat*cgatgt*gtgactggagttcagacgtgtgctcttccgatctgtcaatgatggccggtggatttgtg
AP1_P7_tagged_3	For 2nd hemi-nested PCR of P7 end, binding site to adaptor underlined. Barcode: GCCTAA in italic.	caagcagaagacggcatacgagat*ttaggc*gtgactggagttcagacgtgtgctcttccgatctgtcaatgatggccggtggatttgtg
AP1_P7_tagged_4	For 2nd hemi-nested PCR of P7 end, binding site to adaptor underlined. Barcode: TGGTCA in italic.	caagcagaagacggcatacgagat*tgacca*gtgactggagttcagacgtgtgctcttccgatctgtcaatgatggccggtggatttgtg
AP1_P7_tagged_5	For 2nd hemi-nested PCR of P7 end, binding site to adaptor underlined. Barcode: CACTGT in italic.	caagcagaagacggcatacgagat*acagtg*gtgactggagttcagacgtgtgctcttccgatctgtcaatgatggccggtggatttgtg
AP1_P7_tagged_6	For 2nd hemi-nested PCR of P7 end, binding site to adaptor underlined. Barcode: ATTGGC in italic.	caagcagaagacggcatacgagat*gccaat*gtgactggagttcagacgtgtgctcttccgatctgtcaatgatggccggtggatttgtg
AP1_P7_tagged_7	For 2nd hemi-nested PCR of P7 end, binding site to adaptor underlined. Barcode: CATAGC in italic.	caagcagaagacggcatacgagat*gctatg*gtgactggagttcagacgtgtgctcttccgatctgtcaatgatggccggtggatttgtg
AP1_P7_tagged_8	For 2nd hemi-nested PCR of P7 end, binding site to adaptor underlined. Barcode: CTAGCT in italic.	caagcagaagacggcatacgagat*agctag*gtgactggagttcagacgtgtgctcttccgatctgtcaatgatggccggtggatttgtg
AP1_P7_tagged_9	For 2nd hemi-nested PCR of P7 end, binding site to adaptor underlined. Barcode: TTCGAC in italic.	caagcagaagacggcatacgagat*gtcgaa*gtgactggagttcagacgtgtgctcttccgatctgtcaatgatggccggtggatttgtg
AP1_P7_tagged_10	For 2nd hemi-nested PCR of P7 end, binding site to adaptor underlined. Barcode: CTCGTA in italic.	caagcagaagacggcatacgagat*tacgag*gtgactggagttcagacgtgtgctcttccgatctgtcaatgatggccggtggatttgtg

for native Tn-seq with *E. coli*

*insH3*_out	For 1st PCR, binds near to the 5'end of *insH3*	gataacgccttaaatggcgaagaaac

Staggered_1	For 2nd hemi-nested PCR of P5 end, binding site to *insH3* underlined. T in italic added for heterogeneity.	aatgatacggcgaccaccgagatctacactctttccctacacgacgctcttccgatc*t*gggagaaaaaatcggctcaaacatg
Staggered_2	For 2nd hemi-nested PCR of P5 end, binding site to *insH3* underlined. Ts in italic added for heterogeneity.	aatgatacggcgaccaccgagatctacactctttccctacacgacgctcttccgatc*tt*gggagaaaaaatcggctcaaacatg
Staggered_3	For 2nd hemi-nested PCR of P5 end, binding site to *insH3* underlined. TGATA in italic added for heterogeneity.	aatgatacggcgaccaccgagatctacactctttccctacacgacgctcttccgatc*tgata*gggagaaaaaatcggctcaaacatg
Staggered_4	For 2nd hemi-nested PCR of P5 end, binding site to *insH3* underlined. TATCTA in italic added for heterogeneity.	aatgatacggcgaccaccgagatctacactctttccctacacgacgctcttccgatc*tatcta*gggagaaaaaatcggctcaaacatg

SUPPLEMENTARY REFERENCES

1. van der Valk, R. A. *et al.* Mechanism of environmentally driven conformational changes that modulate H-NS DNA-Bridging activity. *Elife* **6**, e27369 (2017).

2. Abramson, J. *et al.* Accurate structure prediction of biomolecular interactions with AlphaFold 3. *Nature* **630**, 493–500 (2024).

3. Jacobs,A.C., Thompson,M.G., Black,C.C., Kessler,J.L., Clark,L.P., McQueary,C.N., Gancz,H.Y., Corey,B.W., Moon,J.K., Si,Y., *et al.* (2014) AB5075, a highly virulent isolate of acinetobacter baumannii, as a model strain for the evaluation of pathogenesis and antimicrobial treatments. *MBio,* **5**.

4. Gallagher,L.A., Ramage,E., Weiss,E.J., Radey,M., Hayden,H.S., Held,K.G., Huse,H.K., Zurawski,D. V., Brittnacher,M.J. and Manoil,C. (2015) Resources for genetic and genomic analysis of emerging pathogen Acinetobacter baumannii. *J. Bacteriol.*, **197**, 2027–2035.

5. Page,L., Griffiths,L. and Cole,J.A. (1990) Different physiological roles of two independent pathways for nitrite reduction to ammonia by enteric bacteria. *Arch. Microbiol.*, **154**, 349–354.

6. Blattner,F.R., Plunkett,G., Bloch,C.A., Perna,N.T., Burland,V., Riley,M., Collado-Vides,J., Glasner,J.D., Rode,C.K., Mayhew,G.F., *et al.* (1997) The complete genome sequence of Escherichia coli K-12. *Science,* **277**, 1453–62.

7. Lucidi,M., Visaggio,D., Prencipe,E., Imperi,F., Rampioni,G., Cincotti,G., Leoni,L. and Visca,P. (2019) New Shuttle Vectors for Real-Time Gene Expression Analysis in Multidrug-Resistant Acinetobacter Species: In Vitro and In Vivo Responses to Environmental Stressors. *Appl. Environ. Microbiol.,* **85**.

8. Godeux,A.S., Svedholm,E., Lupo,A., Haenni,M., Venner,S., Laaberki,M.H. and Charpentier,X. (2020) Scarless Removal of Large Resistance Island AbaR Results in Antibiotic Susceptibility and Increased Natural Transformability in Acinetobacter baumannii. *Antimicrob. Agents Chemother.*, **64**.
