# Supplementary material for: H-NS is a bacterial transposon capture protein
Source: Nat Commun. 2024 Aug 20;15:7137. doi: 10.1038/s41467-024-51407-5 (PMC11335895; doi:10.1038/s41467-024-51407-5)
Supplement: Supplementary file 3 — Description of Additional Supplementary Files [file 41467_2024_51407_MOESM3_ESM.pdf]

Description of Additional Supplementary Files:

Supplementary Data 1: ChIP-seq read depths

Supplementary Data 2: 3C-seq contact matrices

Supplementary Data 3: 3C-seq explorable heatmaps with aligned ChIP-seq data

Supplementary Data 4: 3C-seq explorable heatmaps with H-NS-39 expression and aligned ChIP-seq data

Supplementary Data 5: ISAba13 insertion sites
