# Supplementary material for: H-NS is a bacterial transposon capture protein
Source: Nat Commun. 2024 Aug 20;15:7137. doi: 10.1038/s41467-024-51407-5 (PMC11335895; doi:10.1038/s41467-024-51407-5)
Supplement: Supplementary file 9 — Reporting Summary [file 41467_2024_51407_MOESM9_ESM.pdf]

nature portfolio

Corresponding author(s): David Grainger

Last updated by author(s): Jul 16, 2024

# Reporting Summary

Nature Portfolio wishes to improve the reproducibility of the work that we publish. This form provides structure for consistency and transparency in reporting. For further information on Nature Portfolio policies, see our Editorial Policies and the Editorial Policy Checklist.

## Statistics

For all statistical analyses, confirm that the following items are present in the figure legend, table legend, main text, or Methods section.

| n/a | Confirmed | |
|---|---|---|
| ☐ | ☒ | The exact sample size (*n*) for each experimental group/condition, given as a discrete number and unit of measurement |
| ☐ | ☒ | A statement on whether measurements were taken from distinct samples or whether the same sample was measured repeatedly |
| ☐ | ☒ | The statistical test(s) used AND whether they are one- or two-sided<br>*Only common tests should be described solely by name; describe more complex techniques in the Methods section.* |
| ☒ | ☐ | A description of all covariates tested |
| ☒ | ☐ | A description of any assumptions or corrections, such as tests of normality and adjustment for multiple comparisons |
| ☐ | ☒ | A full description of the statistical parameters including central tendency (e.g. means) or other basic estimates (e.g. regression coefficient) AND variation (e.g. standard deviation) or associated estimates of uncertainty (e.g. confidence intervals) |
| ☐ | ☒ | For null hypothesis testing, the test statistic (e.g. *F*, *t*, *r*) with confidence intervals, effect sizes, degrees of freedom and *P* value noted<br>*Give P values as exact values whenever suitable.* |
| ☒ | ☐ | For Bayesian analysis, information on the choice of priors and Markov chain Monte Carlo settings |
| ☒ | ☐ | For hierarchical and complex designs, identification of the appropriate level for tests and full reporting of outcomes |
| ☐ | ☒ | Estimates of effect sizes (e.g. Cohen's *d*, Pearson's *r*), indicating how they were calculated |

*Our web collection on statistics for biologists contains articles on many of the points above.*

## Software and code

Policy information about availability of computer code

| | |
|---|---|
| Data collection | Gel images were collected with Quantity One (Biorad) software. |
| Data analysis | Gel images were analysed with Quantity One (Biorad) software. Sequence reads (FASTQ format) were mapped using Bowtie 2 (Galaxy Version 2.4.2) (ChIP-seq, RNA-seq and native Tn-seq) or processed using the HiCup pipeline (Galaxy Version 2.4.2) (3C-seq). Resulting Binary Alignment Map (BAM) files from ChIP-seq and native Tn-seq experiments were visualised using the Artemis genome browser. To map sites of ISAba13 insertion, FASTQ files were parsed using Barcode Splitter (Galaxy version 1.0.1) to identify reads having the final 83 nt of ISAba13 at the 3' end. To account for differences introduced by the staggered oligonucleotides we allowed up to 4 mismatches and 2 deletions. Once selected, the first 83 nt of each read was removed using dada2:filterAndTrim (Galaxy Version 1.20). Only remaining reads 20 or more nt in length were retained. These were then mapped to the reference genome. Read depths for each DNA strand were then calculated using the BAM to Wiggle function of the RSeQC package (Galaxy Version 5.0.1). Subsequent analysis of wiggle files, to identify sites of transposition, was done using logic functions in Microsoft Excel 2016. For differential gene expression analysis of RNA-seq data coverage was first extracted using the genomcov function of BedTools (Galaxy version 2.30.0). FeatureCounts (version 2.6) of the Rsubread (version 2.6) package was used to determine gene read counts, which were inputted into the exact function of edgeR (Galaxy version 3.34.0). HiCup BAM output files were converted to JuiceBox format using hicup2juicer (Galaxy Version 0.9.2). Contact matrices were then generated using OriginLab 2024 and visualised in Microsoft Excel 2016. For whole genome sequencing, Kraken was used to identify a reference genome and reads mapped using BWA mem. Assembly was performed with SPAdes and reads mapped back to contigs with BWA mem. |

For manuscripts utilizing custom algorithms or software that are central to the research but not yet described in published literature, software must be made available to editors and reviewers. We strongly encourage code deposition in a community repository (e.g. GitHub). See the Nature Portfolio guidelines for submitting code & software for further information.

## Data

Policy information about availability of data

All manuscripts must include a data availability statement. This statement should provide the following information, where applicable:

- Accession codes, unique identifiers, or web links for publicly available datasets
- A description of any restrictions on data availability
- For clinical datasets or third party data, please ensure that the statement adheres to our policy

ArrayExpress accession codes: E-MTAB-13791, E-MTAB-13792, E MTAB-13793, E-MTAB-13790 and E-MTAB-13800.
GenBank accession codes: CP144559, CP144563, CP144560, CP144562, CP144564, CP144565

DATA AVAILABILITY STATEMENT

All Illumina sequencing data generated in this study have been deposited in ArrayExpress under accession codes: E-MTAB-13791, E-MTAB-13792, E MTAB-13793, E-MTAB-13790 and E-MTAB-13800. Genome sequences are available from GenBank with accession codes: CP144559, CP144563, CP144560, CP144562, CP144564 and CP144565. Results generated from processing of Illumina sequencing data (e.g. differential gene expression analysis) are available as Source Data or Supplementary Data files. Original gel images are provided as Source Data.

## Research involving human participants, their data, or biological material

Policy information about studies with human participants or human data. See also policy information about sex, gender (identity/presentation), and sexual orientation and race, ethnicity and racism.

| | |
|---|---|
| Reporting on sex and gender | N/A |
| Reporting on race, ethnicity, or other socially relevant groupings | N/A |
| Population characteristics | N/A |
| Recruitment | N/A |
| Ethics oversight | N/A |

Note that full information on the approval of the study protocol must also be provided in the manuscript.

# Field-specific reporting

Please select the one below that is the best fit for your research. If you are not sure, read the appropriate sections before making your selection.

☒ Life sciences ☐ Behavioural & social sciences ☐ Ecological, evolutionary & environmental sciences

For a reference copy of the document with all sections, see nature.com/documents/nr-reporting-summary-flat.pdf

# Life sciences study design

All studies must disclose on these points even when the disclosure is negative.

| | |
|---|---|
| Sample size | Sample sizes were chosen to permit appropriate downstream statistical testing and in line with normal expectations for the field. |
| Data exclusions | No data were excluded. |
| Replication | The RNA-seq, ChIP-seq, native Tn-seq and 3C-seq experiments were done twice to confirm reproducibility. Biochemical experiments (DNA binding and DNA bridging assays) were done at least twice to confirm reproducibility. Microbiological phenotyping experiments were done at least three times. All experiments were reproducible in line with norms for the field. |
| Randomization | Not applicable since samples were not allocated to experimental groups. |
| Blinding | Not applicable since samples were not allocated to experimental groups. |

# Reporting for specific materials, systems and methods

We require information from authors about some types of materials, experimental systems and methods used in many studies. Here, indicate whether each material, system or method listed is relevant to your study. If you are not sure if a list item applies to your research, read the appropriate section before selecting a response.

## Materials & experimental systems

| n/a | Involved in the study |
|---|---|
| ☐ | ☒ Antibodies |
| ☒ | ☐ Eukaryotic cell lines |
| ☒ | ☐ Palaeontology and archaeology |
| ☒ | ☐ Animals and other organisms |
| ☒ | ☐ Clinical data |
| ☒ | ☐ Dual use research of concern |
| ☒ | ☐ Plants |

## Methods

| n/a | Involved in the study |
|---|---|
| ☐ | ☒ ChIP-seq |
| ☒ | ☐ Flow cytometry |
| ☒ | ☐ MRI-based neuroimaging |

# Antibodies

Antibodies used: anti-FLAG (Sigma-Aldrich, catalogue number F7425-2MG)

Validation: Commercial antibody (Sigma-Aldrich) widely used in ChIP-seq experiments.

# Plants

Seed stocks: *Report on the source of all seed stocks or other plant material used. If applicable, state the seed stock centre and catalogue number. If plant specimens were collected from the field, describe the collection location, date and sampling procedures.*

Novel plant genotypes: *Describe the methods by which all novel plant genotypes were produced. This includes those generated by transgenic approaches, gene editing, chemical/radiation-based mutagenesis and hybridization. For transgenic lines, describe the transformation method, the number of independent lines analyzed and the generation upon which experiments were performed. For gene-edited lines, describe the editor used, the endogenous sequence targeted for editing, the targeting guide RNA sequence (if applicable) and how the editor was applied.*

Authentication: *Describe any authentication procedures for each seed stock used or novel genotype generated. Describe any experiments used to assess the effect of a mutation and, where applicable, how potential secondary effects (e.g. second site T-DNA insertions, mosiacism, off-target gene editing) were examined.*

# ChIP-seq

## Data deposition

☒ Confirm that both raw and final processed data have been deposited in a public database such as GEO.

☐ Confirm that you have deposited or provided access to graph files (e.g. BED files) for the called peaks.

Data access links
*May remain private before publication.*: The raw data are assigned accession code E-MTAB-13790 and are available from ArrayExpress.

Files in database submission: Raw reads in FASTQ format

Genome browser session (e.g. UCSC): N/A

## Methodology

Replicates: Two replicates

Sequencing depth: See FASTQ files

Antibodies: Anti-FLAG (Sigma)

Peak calling parameters: N/A (H-NS does not form discrete peaks but binds over large regions).

Data quality: Visual inspection of coverage and alignment of H-NS binding signals with AT-tich DNA sequences.

Software: Reads were mapped using Bowtie 2 and resulting Binary Alignment Map (BAM) files visualised using the Artemis genome browser.
