# Supplementary figures and images for: H-NS is a bacterial transposon capture protein
Source: Nat Commun. 2024 Aug 20;15:7137. doi: 10.1038/s41467-024-51407-5 (PMC11335895; doi:10.1038/s41467-024-51407-5)

Figure 4b

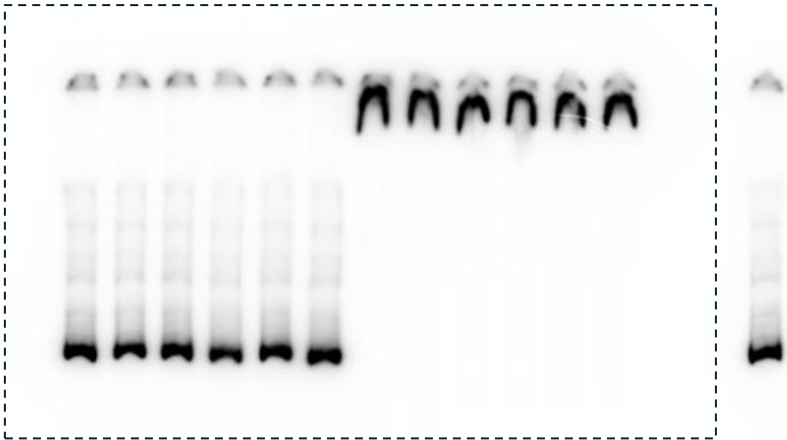

Supplement: Supplementary file 10 — Source data [file 41467_2024_51407_MOESM10_ESM.zip › Source data uncropped gels.pdf]
